# Case of Oedema of the Glottis, Following Tonsillitis.—Laryngotomy, &c.

**Published:** 1845-07-01

**Authors:** James P. White


### Case of Oedema of the Glottis, following Tonsillitis.—Laryngotomy, &c.

Communicated for the Buffalo Medical Journal, by JAMES P. WHITE, M. D.

M. L. F. auctioneer, aged 39, spare man, of active habits, sent for me on the evening of March 6th; found him laboring under tonsillitis, and learned that two days previous he had taken cold by sitting most of the day near an open window; that sore throat had succeeded, and that he had taken senna and salts in operative doses, and made use of some mild astringent gargle. The attack was accompanied with considerable febrile irritation, and moderate local swelling, but was not thought dangerous; indeed, I was sent for rather to prescribe for the daughter, who was suffering with the same affection. The pulse was 100, not very hard or full; thirst considerable, drinks taken without much difficulty; cathartic had operated during the day; skin dry and hot. Directed him to rub the surface of the neck with the following mixture. ℞ camphorated oil aquae ammoniae *aa* ℥i. mix; and as he had slept little the night previous, gave him three perspirative anodyne powders, one to be taken every 3 or 4 hours, until sleep should be procured.

7th, morning—had taken all the powders, slept well, felt refreshed, pulse 84, skin more moist and cool, and swelling of tonsils not increased. Directed the following recipe: sup. tart potass ʒii., tart antim. et potass gr. iv., mix; to be dissolved in one pint hot water, and take a wine glass every two hours. 6 P. M.—had conversed during the day considerably relative to business, and throat was more painful, fever increased, tonsils reddened and increased in size. Scarified the tonsils, directed the antimony and crem. tartar, which had been neglected during the day, to be increased so as to produce nausea, and interdicted all conversation. Saturday, 8 A. M. fever much diminished, but throat sore on pressure externally, and deglutition painful. Scarified the tonsils, applied 8 leeches to the neck, continued the solution, and moved the bowels with calomel, followed by senna and salts. 6 P. M. Bowels had moved freely, skin moist, pulse much softer, and patient expressed himself as feeling "much better but languid." Tonsils less red and painful, deglutition not very difficult, and respiration not at all interfered with. Directed him to take the anodyne, as on Thursday night.

9th, 5 A. M.—Was called out of bed in great haste, being told that the patient was dying. On my arrival, found him unconscious, face engorged with unarterialised blood, breathing nearly ceased, loud and spasmodic. I immediately raised his head, thrust the handle of a spoon between the tonsils, to ascertain if they were so large as to produce suffocation, but found them so far separated as to permit the spoon to be turned flatwise without much difficulty. At this time, the larynx and trachea were pumping like the piston of a steam-engine at each inspiration, the expiration being free, but accompanied by a flapping, stertorous sound. I immediately suspected effusion into the cellular tissue at the top of the glottis, and proceeded to expose the Crico-thyroid space, believing the operation of Laryngotomy afforded the only chance of relief, although this was but faint. By this time my friend Dr. Flint, whose residence was next adjoining, came in, when we inserted an elastic tube, and inflated the lungs, making alternate efforts of blowing into the trachea and compressing the chest, but to no purpose, the patient made not a single effort to breathe after the operation.

I learned that he took one of the Dovers powders at 6 and 9 P. M. and passed a comfortable evening; took the last powder at 1 A. M.; took drink freely and slept well until at 2½, when his wife being somewhat alarmed by his loud breathing aroused him. He took at this time half a tumbler of toast water without difficulty, but remarked that his throat felt strangely, that the swelling seemed extending downward. He put his hand on the pomum adami, saying it was there, and thrust his finger quite down into the throat in an effort to feel the source of the difficulty. He soon, however, went again to sleep, and Mrs. F. whose rest had been much disturbed, also fell into a pretty sound sleep, from which she was awakened by his loud and stertorous breathing, and on going to him found she could not arouse him.

---

POST MORTEM EXAMINATION THREE DAYS AFTER DEATH; Present Drs. Josiah Trowbridge, Charles Winne, and Austin Flint. On examining the larynx, the mucus membrane of the glottis was found greatly distended with serum, forming large bladders, and so occupying the rima glottidis as to have completely closed the aperture during inspiration.

Tonsils not greatly enlarged. Mucus membrane of the fauces of a dull red colour, in the trachea healthy.

The practical deductions to be drawn from the above case are, that it is unsafe even in a mild case of Quinsy, to leave patients without some attendant awake beside them, as has heretofore been the habit of all the profession, especially during the subsidence of the active symptoms; and that in a case which becomes complicated like the one just related, the operation of Laryngotomy, if resorted to at the proper period, would offer a tolerable chance of success.

My friend, Prof. De LaMater, of Cleveland, relates to me an instance which occurred a few years since, in which he operated successfully, although the patient seemed in articulo mortis at the time. It would seem, however, important to success, that the operation should be performed before the brain or lungs have suffered lesion.

*Buffalo, May* 27, 1845.

